# Meta-analysis data quantifying nitrous oxides emissions from Chinese vegetable production

**DOI:** 10.1016/j.dib.2018.05.034

**Published:** 2018-05-11

**Authors:** Xiaozhong Wang, Xiaopeng Gao, Xinping Chen

**Affiliations:** a Academy of Agricultural Sciences, Southwest University, Chongqing 400716, China; b College of Resources and Environment, Southwest University, Chongqing 400716, China; c Center for Resources, Environment and Food Security, China Agricultural University, Beijing 100193, China; d State Key Laboratory of Desert and Oasis Ecology, Xinjiang Institute of Ecology and Geography, Chinese Academy of Sciences, Urumqi 830011, China

**Keywords:** N_2_O, nitrous oxides, N, nitrogen, EFs, emission factors, RR, the response ratio, Chinese vegetable fields, Meta-analysis, Nitrous oxide emission, Emission factor

## Abstract

This paper describes data of nitrous oxides (N_2_O) emissions from open-field and greenhouse systems in Chinese vegetable production. The data also describes the potential soil and management factors to identify the effective measures to mitigate N_2_O emissions. The data were collected from 21 peer-reviewed papers, covering 153 N_2_O emission field measurements as affected by fertilizer nitrogen (N) management. This data were subjected to meta-analysis for a comprehensive assessment on N_2_O emission and applied N based emission factor in Chinese vegetable production.

**Specifications Table**

**Table:** 

Subject area	*Agriculture*
More specific subject area	*Agricultural environmental pollution*
Type of data	*Table*
How data was acquired	*Data was acquired from the published articles*
Data format	*Raw, analyzed*
Experimental factors	*The criteria used for screening previous publications were described in Wang et al.*[1].
Experimental features	*Data were subjected to meta-analysis to quantify nitrous oxides (N*_*2*_*O) emissions in Chinese vegetable production and identify the effective fertilizer management practices to reduce emissions.*
Data source location	*China*
Data accessibility	*Data are within this article*
Related research article	*Wang et al.*[1]

**Value of the data**

• Data cover 153 field measurements of N_2_O emission from 21 experiments.
• Data are suitable for meta-analysis.
• Data can be used to quantify N_2_O emissions and emission factors (EFs) from Chinese vegetable production in open-field and greenhouse systems.
• Data can be used to identify effects of fertilizer N managements (rate and type) and soil properties (Soil organic matter, total N content, etc.) on N_2_O emissions and EFs.

## 1 Data

Data were extracted from peer-reviewed journal papers published until 2017 January. Totally 153 field measurements of N_2_O emission from 21 experiments were included in a meta-analysis. Detailed data are listed in supplementary material, giving the following information: year, location, vegetable species, cultivation type, formulation of synthetic and manure N fertilizer, N fertilizer rate, cumulative N_2_O emission over the growing season, EFs, the response ratio (RR), yield, soil organic matter content, soil total N content, irrigation, rainfall, duration of experimental period, gas sampling method, literature reference.

## 2 Experimental design, materials, and methods

### 2.1 Design, materials, and methods

Databases including ISI-Web of Science (Thomson Reuters, New York, NY, USA), Google Scholar (Google Inc., Mountain View, CA, USA), and the China Knowledge Resource Integrated database (CNKI) were used to search for studies enrolled in the meta-analysis. The Keyword search included ‘nitrous oxide’, ‘N_2_O’, ‘nitrogen’, and ‘vegetable’, which were adopted to identify all relevant literatures on N_2_O emissions from vegetable production. Data in only graphical form were digitized using GetData v.2.22 software [2].

To minimize bias, the following criteria were used to select studies for meta-analysis: (i) N_2_O emissions were measured during the entire growing seasons of vegetable crops in open-field or greenhouse systems, and the vegetables were grown in the field rather than in soil columns or pots; (ii) N_2_O emissions were reported for experiments that included treatments both with and without N fertilizer application, to calculate the EFs; (iii) N fertilizer sources were synthetic fertilizer or manure (e.g. pig, cattle, poultry manures, plant residues, compost etc), but the synthetic fertilizer could only include conventional commercial N fertilizer (e.g., urea, thiamine, compounds), i.e., could not include slow-/controlled-release or stabilized fertilizers.; (iv) N_2_O emissions were measured with manually operated chambers, automatic chambers, or towers.

### 2.2 Meta-analysis

An unweighted pair-wise meta-analysis were conducted on N_2_O emission and EFs. The effect size for each control-treatment pair was estimated using the response ratio [RR=ln(N_2_O_treatment_/N_2_O_control_), where N_2_O_treatment_ and N_2_O_control_ were means of N_2_O emissions (kg N_2_O-N ha^−1^) for the fertilized treatment and for the unfertilized control in each field observation, respectively [3]. Data of RR, N_2_O emission and EFs were subjected to Kolmogorov–Smirnov test for determination of normal distribution [4]. Response ratio followed normal distribution, suggesting the capacity of using meta-analysis for examination of fertilizer N effects on N_2_O emissions. Data of N_2_O emission and EFs followed non-normal distribution and were therefore subjected to nonparametric test for further analysis.
