# Supplementary material for: Meta-analysis data quantifying nitrous oxides emissions from Chinese vegetable production
Source: Data Brief. 2018 May 11;19:114–6. doi: 10.1016/j.dib.2018.05.034 (PMC5993008; doi:10.1016/j.dib.2018.05.034)
Supplement: Supplementary file 1 — Supplementary material [file mmc1.pdf]

Declaration of interest

We confirm that the manuscript entitled "Meta-analysis data quantifying nitrous oxides emissions from Chinese vegetable production " is original, has not been full or partly published before, and is not currently being considered for publication elsewhere.

We confirm that there are no known conflicts of interest associated with this publication and there has been no significant financial support for this work that could have influenced its outcome.

We confirm that the manuscript has been read and approved by all named authors and that there are no other persons who satisfied the criteria for authorship but are not listed.

We further confirm that the order of authors listed in the manuscript has been approved by the undersigned.

Signed by all authors as follows:

Xiaopeng Gao (XIAOPENG GAO)

Xinping Chen (XINPING CHEN)

Xiaozhong Wang (XIAOZHONG WANG)
